# Glaucoma suspects referred by general ophthalmologists to a tertiary center in Brazil: outcomes of the glaucoma specialist assessment

**DOI:** 10.5935/0004-2749.20230029

**Published:** 2023

**Authors:** Fernanda Limeira Fanton, Egidio Picetti, Stefanie Ferrari Rodrigues, Thaina Silva Moreira, Rodrigo Leivas Lindenmeyer, Fabio Lavinsky, Helena Messinger Pakter

**Affiliations:** 1 Grupo Hospitalar Conceição, Porto Alegre, RS, Brazil; 2 Hospital de Clínicas de Porto Alegre, Porto Alegre, RS, Brazil; 3 Faculdade de Medicina/Universidade Federal do Rio Grande do Sul, Porto Alegre, RS, Brazil

**Keywords:** Glaucoma/diagnosis, Ocular hypertension, Glaucoma, open-angle/diagnosis, Glaucoma, angle-closure/diagnosis, Tertiary healthcare, Practice patterns, physicians’, Glaucoma/diagnóstico, Hipertensão ocular, Glaucoma de ângulo aberto/diagnóstico, Glaucoma de ângulo fechado/ diagnóstico, Atenção terciária à saúde, Padrões de prática médica

## Abstract

**Purpose:**

To characterize patients with suspected glaucoma who were referred to the clinic for suspected glaucoma in a tertiary public hospital in southern Brazil and to evaluate differences in functional and structural damages between patients diagnosed with different types of glaucoma, those with normal eye examination results, and those who remained as glaucoma suspects.

**Methods:**

This is a cohort study of patients referred by general ophthalmologists to the clinic for suspected glaucoma at Hospital Nossa Senhora da Conceição, Porto Alegre, Brazil, between March 2016 and December 2018. The patients were followed up until they had undergone reliable examinations (eye examination, visual field screening, and optic coherence tomography for classification as normal and having a suspected glaucoma, glaucoma with an elevated intraocular pressure, normotensive glaucoma, or ocular hypertension.

**Results:**

A total of 135 patients were included in this study. Of the patients, 117 subjects completed all examinations and met the inclusion criteria. Most patients were normal (36.8%), followed by those with suspected glaucoma (25.64%), normal tension glaucoma (18.8%), glaucoma with elevated intraocular pressure (12%), and ocular hypertensive (6%). The main reason for referral was increased optic nerve head cupping. The patients with normal tension glaucoma were older than the other subjects on average (p=0.03). In addition, the normal tension glaucoma group had a significantly worse baseline visual field index and mean deviation of the visual field than the normal, glaucoma suspect, and ocular hypertensive groups. The circumpapillary retinal nerve fiber layer on OCT was thinner on average in the normal tension glaucoma group than in the normal and glaucoma suspect groups (p<0.002) but did not significantly differ between the glaucoma group with elevated intraocular pressure and the other groups.

**Conclusions:**

Patients with normal tension glaucoma tend to be diagnosed later because of their normal intraocular pressures; thus, the optic nerve cupping must be greater to raise the suspicion of glaucoma. In this study, we found that the patients with normal tension glaucoma had worse disease at the time of diagnosis.

## Abstract (pt)

**Objetivo:**

Caracterizar a população com suspeita de glaucoma encaminhada a um centro público terciário no sul do Brasil e avaliar diferenças no dano dos parâmetros funcionais e estruturais entre os pacientes diagnosticados com diferentes tipos de glaucoma e aqueles classificados como normais e aqueles mantidos como suspeitos de glaucoma.

**Métodos:**

Esta é uma coorte dos pacientes encaminhados para o setor de glaucoma suspeito do Hospital Nossa Senhora da Conceição, Porto Alegre - BR, no período de março de 2016 a dezembro de 2018. Os pacientes foram acompanhados até obterem exames confiáveis (exame oftalmológico completo, campimetria visual, tomografia de coerência óptica) para serem classificados como: normal, glaucoma suspeito, glaucoma com pressão intraocular elevada, glaucoma de pressão normal ou hipertenso ocular.

**Resultados:**

Um total de 135 pacientes foram incluídos neste estudo, sendo que destes, 117 pacientes completaram todos os exames e foram incluídos neste estudo. A maioria dos pacientes foi considerada normal (36,8%), seguido por glaucoma suspeito (25,64%), glaucoma de pressão normal (18,8%), glaucoma com pressão intraocular elevada (12%) e hipertensão ocular (6%). A principal razão para encaminhamento foi escavação do nervo óptico aumentada. Pacientes com glaucoma de pressão normal eram em média mais velhos que os demais (p=0,03). Esses também apresentavam índice de campo visual e desvio médio da campimetria visual piores que sujeitos normal, com suspeita de glaucoma e hipertensos oculares, e tinham a camada de fibra nervosa medida pela tomografia de coerência óptica mais fina que normais e suspeitos de glaucoma (p<0,002). Os pacientes com glaucoma de pressão elevada não diferiram significativamente dos outros grupos.

**Conclusão:**

Pacientes com glaucoma de pressão normal tendem a ser diagnosticados mais tardiamente devido ao fato da pressão intraocular não estar elevada, logo a escavação do disco óptico deve ser maior para gerar a suspeita de glaucoma. Neste estudo, paciente com glaucoma de pressão normal apresentaram doença mais avançada no momento do diagnóstico em comparação com os outros grupos.

## INTRODUCTION

Glaucoma is a progressive optic neuropathy in which structural modifications on the optic nerve head (ONH), ganglion cell layer, and retinal nerve fiber layer (RNFL) are associated with characteristic visual field (VF) defects^(1,2)^. Owing to its asymptomatic and insidious evolution in its early stages, its diagnosis is usually late.

Sakata et al. found an incidence rate of almost 90% of undiagnosed glaucoma in Brazilian patients aged >40 years. This suggests the necessity for screening and suspicion of glaucoma in the early stages of the disease^(3)^.

A glaucoma suspect is an individual with one or more clinical features and/or risk factors that increase the possibility of developing glaucomatous optic neuropathy (GON) and visual impairment in the future^(4,5,6)^. Glaucoma suspects have one of the following findings: constantly elevated intraocular pressure (IOP; >21 mmHg) and suspicious optic nerve or VFs in the absence of other neuropathies^(7)^.

Little consensus among ophthalmologists and researchers has been reached on how to define glaucoma, glaucoma suspects, and ocular hypertension. For instance, some ophthalmologists require both VF loss and evidence of GON for a diagnosis of glaucoma, referring to those with only one of these findings as glaucoma suspects. Others consider the presence of GON, with or without repeatable VF loss, as sufficient to diagnose glaucoma. That is the difficulty in the diagnosis of glaucoma, especially in populations without consistent VF loss. Despite this lack of consensus on how to define glaucoma, most experts agree that the presence of structural damage is required, whereas functional loss measured by visual perimetry can be used to increase the likelihood of the disease or confirm the diagnosis^(8)^.

To evaluate structural optic nerve and retinal changes, optical coherence tomography (OCT) is used for objective and quantitative measurements of the thicknesses of the retinal nerve fiber and ganglion cell layers and other ONH parameters. To evaluate functional damage, the automated perimetry remains the gold standard for diagnosis and evaluation of the progression of the glaucomatous damage. Studies have shown that the detection of structural damage often precedes the detection of functional damage, and technologies that measure the thickness of the circumpapillary RNFL (cRNFL) are more sensitive in detecting the disease earlier than the VF test^(9)^. However, probably the best strategy for early diagnosis involves the combination of the information from functional and structural tests^(10,11)^.

In developed countries, population studies have shown that only half of glaucoma patients are aware of the disease.^(12,13)^ In Brazil, information is lacking, and the real extent of vision-related problems is unknown. Approximately 2%-3% of the population aged >40 years have primary open-angle glaucoma, but identifying glaucoma suspects and correctly diagnosing them are even greater challenges^(14)^.

In this context, the aim of this study was to characterize the glaucoma suspect population referred to a glaucoma suspect clinic of a tertiary public hospital in southern Brazil. In addition, we evaluated differences in functional and structural damages between patients diagnosed with different types of glaucoma who remained as glaucoma suspects and those who were considered normal.

## METHODS

### Participants

This is a cohort study of patients referred by general ophthalmologists to the glaucoma referral center of Hospital Nossa Senhora da Conceição Hospital between March 2016 and December 2018. The study was approved by the research ethics committee of the Grupo Hospitalar Conceição and followed the tenets of the Declaration of Helsinki.

The patients were followed up until they had undergone reliable examinations (eye examination, VF screening, and OCT) for classification as normal, glaucoma suspects, having glaucoma with elevated intraocular pressure (IOP), having normotensive glaucoma (NTG), or having ocular hypertension (OH).

### Inclusion criteria

Patients aged ≥18 years who were considered as glaucoma suspects by general ophthalmologists were invited to participate. In general, they had to have at least one of the following: elevated IOP on at least one eye (>21 mmHg), previous history of elevated IOP, apparently suspicious ON, and/or suspicious VF referred to the glaucoma suspect clinic.

### Exclusion criteria

Patients were excluded if they had already been diagnosed as having glaucoma, were using eye drops for the treatment of glaucoma, and already undergone a previous laser or surgery for glaucoma. Patients were also excluded if they had a 20/50 vision or worse in either eye or severely affected by other eye diseases (i.e., trauma and central retinal vein occlusion) due to loss of the possibility for comparison with the fellow eye.

### Ophthalmic examination

The patients underwent complete ophthalmologic examination, including best-corrected visual acuity with Snellen optotypes, anterior segment evaluation using a slit lamp (searching for corneal pathologies or other characteristics that could be related to IOP increase and risk of development of glaucoma such as pseudoexfoliation material, pigmentary dispersion, iris and angle neovascularization, and inflammation), IOP measurement using a Perkins applanation tonometer, gonioscopy using a Zeiss 4-mirror lens, and fundus examination under mydriasis.

Individuals with at least three IOP measurements (in different visits) were classified as having ocular hypertension (IOP of 22 mmHg or greater) or normal tension (IOP<22 mmHg).

Central corneal thickness measurement anterior segment tomography (Oculus Pentacam HR) was performed in all the patients, as IOP measurement can be falsely elevated or diminished depending on the corneal thickness.^(15)^

Automated perimetry was performed (Zeiss Humphrey, SITA 24-2). As defined by Bernardi et al., examination results were considered eligible if false negatives and false positives were <33% and the fixation loss was <20%^(16)^. The VF was considered abnormal according to the Hodapp, Parrish, and Anderson’s criteria if the pattern standard deviation was <5%, the glaucoma Hemifield test result is outside the normal limits, or three contiguous points are present with a sensibility of <5% and one of which is depressed at p<1%^(4)^.

Digital color retinography was performed using the Visucam Fundus Camera (Zeiss) and was evaluated by a glaucoma expert. The evaluation included estimated vertical and horizontal cup-to-disk ratios, the neuro-optical rim and ISNT (inferior ≥ superior ≥ nasal ≥ temporal) rule (the anatomical relation of the rim on each sector of the optic disk, with the inferior being the largest and the temporal being the thinnest)^(17)^, and the presence of RNFL defects (*Hoyt*) or disk hemorrhages. Suspected glaucomatous optic neuropathy (SGON) was defined as optic nerve cupping (vertical cup-to-disk ratio [VCDR] >0.5), neuroretinal rim thinning or notching, localized or diffuse RNFL defect, or VCDR asymmetry >0.2 between the eyes (not explained by differences in disk size), on the basis of masked grading by EP^(8)^.

cRNFL was measured using the OCT Cirrus HD Spectral Domain V6.5 software. The eyes were classified using the OCT software as normal, abnormal, or borderline. The mean cRNFL was also recorded.

The patients were followed up until they had undergone reliable examinations for classification as follows:

Primary open-angle glaucoma (POAG): eyes with open angles and glaucomatous optic neuropathy (GON) and/or RNFL defects/thinning with clinical repercussion on the VF and IOPs >21 mmHg measured at any medical visit.

Normotensive glaucoma (NTG): GON and/or RNFL defects/thinning with clinical repercussions on the VF and IOPs <21 mmHg.

Primary angle-closure suspect: iridotrabecular contact (closed angle) for at least 180° on gonioscopy, no peripheral anterior synechiae (PAS), normal IOP, and no evidence of GON.

Primary angle closure: iridotrabecular contact (closed angle) for at least 180° on gonioscopy, with either PAS or raised IOP, and no evidence of GON.

Primary angle-closure glaucoma (PACG): complete or partial closure of the anterior chamber angle due to apposition of the peripheral iris to the posterior cornea in >180° with or without the presence of PAS and/or iris imprinting and in the presence of increased IOP and GON and/or VF loss.

Secondary glaucoma: eyes with GON and/or RNFL defects/thinning with clinical repercussion on VF and IOPs >21 mmHg measured on any medical visit due to other ocular disease.

Ocular hypertensives (OH): IOPs >21 mmHg, regardless of central corneal thickness, without SGON and with normal VF or with VF changes not suggestive of glaucoma.

Patients who had normal results in most examinations or minimal changes not consistent with glaucoma were considered normal. The remaining patients who did not meet the criteria for the disease but appeared to be at risk of developing glaucoma were continued to be followed up as glaucoma suspects.

To further compare the outcomes, we grouped the patients as follows:

1. Normal group: patients with eye examination results within the normal limits and were discharged.
2. Glaucoma suspect group: patients who were not considered as having glaucoma at this time, but the diagnosis could not be excluded; thus, follow up was continued.
3. Glaucoma with elevated IOP group: patients with glaucoma or very likely to have glaucoma in the presence of an IOP >21 mmHg; treatment was initiated. The etiology in this group could be POAG or PACG.
4. NTG group: patients with glaucoma or very likely to have glaucoma, with an IOP <21 mmHg; treatment was initiated.
5. Ocular hypertension group: patients diagnosed with OH and followed up or treated in accordance with other risk factors.

In general, the patients required multiple visits and examinations to confirm or rule out the diagnosis of glaucoma. We used data from the most recent or most reliable examination.

### Data analysis

Generalized estimating equations, which consider the correlation between both eyes of the same individual, were used to compare the structural and functional parameters between the groups. We used binomial distribution and logit function for categorical data and normal distribution to identity the association with continuous data. Continuous variables are described as mean and standard deviation (SD). Statistical significance was defined at p<0.05.

The intraclass correlation coefficient (ICC) was used to test the agreement between the vertical cup-to-disc ratio as assessed by glaucoma specialists on disc photographs and the cup-to-disc ratio described by the general ophthalmologist based on fundus biomicroscopy images at the referral.

Statistical analyses were performed using the SPSS version 18 software.

## RESULTS

A total of 145 patients were referred to the glaucoma suspect clinic. Of these patients, 117 completed all the examinations and met the inclusion criteria. The remaining were not enrolled because they had other diagnoses (mainly neurological diseases or retinal scars) or did not finish the examinations (Table 1).

**Table 1:** Diagnostic categories of all the patients referred to the glaucoma suspect clinic

	Number of patients	Percentage
**Normal**	43	29.7%
**Open-angle glaucoma suspect**	29	20%
**Closed-angle glaucoma suspect**	1	0.7%
**Ocular hypertensive**	7	4.8%
**Primary open-angle glaucoma**	10	6.9%
**Primary angle-closure glaucoma**	4	2,8
**Secondary glaucoma**	1	0.7%
**Normotensive glaucoma**	22	15.2%
**Subtotal**	(117)	(80.7%)
**Other ocular/systemic diseases**	15	10.3%
**Lost to follow-up**	12	8.3%
**Death**	1	0.7%
**Total**	145	100%

The participants’ ages ranged from 21 to 79 years (mean, 58.2 ± 11 years) and significantly differed between the groups (analysis of variance least significant difference, p=0.03). The patients in the NTG group were older (64.36 ± 8.91 years) than those in the other diagnostic groups on average. Most patients were female (74.4%), and female sex was predominant in all the diagnostic groups, except in the NTG group, which had equal proportions of sex. This population was mostly composed of whites (76.1%). Other races were similarly distributed between the groups (15.4% blacks and 7.7% brown), and only one Asian was diagnosed as having NTG (Table 2).

**Table 2:** Demographic and clinical characteristic of the subjects included in study

	Normal	Glaucoma suspect	Glaucoma	Normotensive glaucoma	Ocular hypertension
**Basic characteristics of the study individuals**					
**Frequency**	43 (36.8%)	30 (25.64%)	15 (12.8%)	22 (18.80%)	7 (6%)
**Age**	56.74 ± 12.3	58.16 ± 6.7	55.86 ± 11.9	**64.36 ± 8.9** 1	52.14 ± 16.1
**Female sex**	34 (79.1%)	25 (83.3%)	12 (80%)	11 (50%)	5 (71.4%)
**White race**	35 (81.4%)	22 (73.3%)	10 (66.7%)	17 (73.3%)	5 (71.4%)
**SAH**	12 (27.9%)	10 (33.3%)	3 (20%)	13 (59.1%)	1 (14.3%)
**DM**	7 (16.3%)	2 (6.7%)	4 (26.7%)	5 (22.7%)	1 (14.3%)
**Reasons of referral**					
**Increased ON cupping, n (%)**	24 (55.8%)	19 (63.3%)	6 (40%)	19 (86.4%)	2 (28.6%)
**Asymmetric ON cupping, n (%)**	6 (14%)	5 (16.7%)	4 (26.7%)	2 (9.1%)	0 (0%)
**Altered visual field**	0 (0%)	2, 6 (7%)	0 (0%)	0 (0%)	0 (0%)
**Increased IOP**	7 (16.3%)	1 (3.3%)	5 (33.3%)	0 (0%)	5 (71.4%)
**Previous diagnosis**	6 (14%)	3 (10%)	0 (0%)	1 (4.5%)	0 (0%)
**Intraocular pressure mmHg (mean** ± **SD)**	14.08 ± 3.9	13.63 ± 2.8	**20.3 ± 4.52**	13.59 ± 2.6	**22.7 ± 1.2** 2
**Central corneal thickness** µ**c (mean** ± **SD)**	537.7 ± 27.8	546.9 ± 34.4	548.1 ± 35.4	541.7 ± 35.5	**573.4 ± 31.7** 3
**Optic coherence tomography nerve fiber layer thickness** µ**c (mean** ± **SD)**	91.71 ± 9.65	90.02 ± 7.68	85.67 ± 8.44	**78.95 ± 8.44** 4	93.36 ± 11.02
**Optic coherence tomography nerve fiber layer (normal, borderline, or abnormal)**					
**Normal**	84 (97.7%)	59 (98.3%)	28 (93.3%)	26 (59.1%)	13 (92.9%)
**Borderline**	2 (2.3%)	1 (1.7%)	2 (6.7%)	10( 22.7%)	1 (7.1%)
**Abnormal**	0 (0%)	0 (0%)	0 (0%)	8 (18.2%)	0 (0%)
**Visual field (normal, borderline, or abnormal)**					
**Normal**	75 (87.2%)	32 (53.3%)	12 (40%)	4 (9.1%)	6 (42.9%)
**Abnormal**	1 (1.2%)	24 (40%)	16 (53.3%)	38 (86.4%)	6 (42.9%)
**Non-reliable**	10 (11.6%)	4 (6.7%)	2 (6.7%)	2 (4.5%)	2 (14.3%)
**Visual field index, % (mean** ± **SD)**	98 ± 3.15	97.20 ± 2.82	95.86 ± 4.56	**91.19 ± 8.42** 5	98.08 ± 1.68
**Visual field mean deviation, dB (mean** ± **SD)**	-1.50 ± 1.23	-1.88 ± 1.78	-2.43 ± 1.93	**-4.46 ± 3.26** 6	-2.05 ± 1.04

SAH= systemic arterial hypertension; DM: diabetes mellitus; ON= optic nerve; IOP= intraocular pressure; SD= standard deviation; mmHg= millimeters of mercury; µc= micrometers; dB= decibels; NTG= normal tension glaucoma; OH= ocular hypertension. The boldface indicate a significant difference.

1. NTG older than all other groups (p=0.03, one way analysis of variance least significant difference).

2. Glaucoma and OH IOP higher than those in all the other groups (p<0.05).

3. OH CCT thicker than that in the normal group p<0.005.

4. NTG NFL thinner than those in the normal and glaucoma suspect groups (p<0.005).

5. NTG VFI worse than those in the normal, glaucoma suspect, and OH groups (p<0.002).

6. NTG MD worse than those in the normal, glaucoma suspect, and OH groups (p<0.004).

Nineteen patients (16.2%) reported diabetes mellitus, and 39 patients (33.3%) were known to have systemic hypertensive. More than half (59%) of the patients with NTG had systemic hypertension. Most patients did not have a positive family history of glaucoma (57.3%) or were unaware of that (17.1%; Table 2).

Nearly 60% of the patients were referred because of increased cupping on ophthalmic examination. In all the diagnostic groups, this was the main reason for referral, followed by cup asymmetry (14.5%), except for OH, whose the main reason for referral was increased IOP (71.4%), as expected (Table 2).

The mean IOP was 15.2 ± 4.4 mmHg (range, 6-28 mmHg). As expected, patients with glaucoma with elevated IOP had a mean IOP greater than those of the normal, glaucoma suspect, and NTG groups but lower than that of the OH group (p<0.05; Table 2).

On average, the central corneal thickness was greater in the OH group (573.4 ± 31.7 µm), but this was only significantly different from that in the normal group (537.7 ± 27.8 µm; p=0.006). The NTG group had thinner CCT (541.7 ± 35.5 µm) than the glaucoma group with elevated IOP (548.1 ± 35.4 µm), glaucoma suspects (546.9 ± 34.4 µm), and OH group (573.4 ± 31.7 µm), but this did not reach statistical significance.

In general, optic nerve evaluation by fundus biomicroscopy tended to be more frequently used to grade eyes with SGON than color retinography (65% vs 45.3%). The ICC agreement between C/D graded by one masked glaucoma expert on color retinographies and an attendant ophthalmologist on fundus biomicroscopy was 0.74 (Cronbach’s alpha, p<0.05). Similar findings have been described previously^(18)^.

Comparing the key points of the changes in OCT and VF, we observed that the NTG group had greater prevalence rates of abnormal OCT and VF results than the glaucoma group with elevated IOP (abnormal OCT nerve fiber laser [NFL], 40.9% vs 6.7% and abnormal VF, 90.9% vs 58.6%).

The Pearson correlation coefficient between the mean OCT NFL and VF MD was 0.32, with insignificance at the 0.01 level (two-tailed). This correlation was a little better in the NTG group (0.47).

The NTG group had the worst mean MD (-4.46dB ± 3.26) than the other groups (normal, -1.50 dB; glaucoma suspects, -1.88 dB; glaucoma with elevated IOP, -2.43 dB; and OH, -2.06 dB; p<0.005 for difference from the normal, GS, and OH groups, and p=0.06 [age adjusted] for the glaucoma group with elevated IOP). This was similar to the VF index (VFI), as the NTG group had the worst mean VFI (VFI, 91.19 ± 8.42), followed by the group with other glaucomas (VFI, 96%; p=0.08), glaucoma suspects (VFI, 97.20% ± 2.82%; p<0.0001), and normal and OH groups, with a mean VFI of 98% (p<0.0001). By using Hodapp, Parrish, and Anderson’s criteria to classify VF defects, patients were often classified as having an abnormal VF (normal, 1.2%; glaucoma suspects, 40%; patients with glaucoma with elevated IOP, 53.3%; NTG, 86.4%; and OH, 42.9%; Table 2).

The mean OCT NFL thickness was thinner in the NTG group (78.9 ± 11 µm), but only significantly different from those in the normal and glaucoma suspect groups (normal, 91.7 ± 9 µm; suspects, 90.0 ± 7 µm; glaucoma with elevated IOP, 85.7 ± 2 µm; OH, 93.4 ± 11.0 µm; p<0.02). By using the OCT software to discriminate between normal, borderline, and abnormal average cRNFL scans, the examinations results considered abnormal were rare and only present in the NTG group (18.2% abnormal and 22.7% borderline; Table 2).

## DISCUSSION

Diagnosis of glaucoma as the main cause of irreversible blindness is critical. Detecting early glaucoma in healthy individuals and glaucoma suspects can be a difficult task for both ophthalmologists and patients, as it usually requires many tests, often at multiple visits. This overall burden is justified if disease progression can be prevented and, at the same time, the overall costs of IOP-lowering therapy can be minimized by reserving the therapy for high-risk glaucoma suspects. In this report, we describe the epidemiological characteristics of a population of glaucoma suspects referred to a tertiary public center and show some important differences between the diagnostic groups.

One fourth of the referred patients were indeed suspects, most of whom had increased *C/D* ratio or suspected ON, normal results in other examinations, or findings not in consonance with those of other studies. These patients are still under our close surveillance.

In this population, NTG was the most common form of glaucoma (19%) despite the fact that epidemiologically POAG is one of the most prevalent types of glaucoma in the Western world^(12)^. This could be related to the fact that patients with unmistakably high IOP and suspected optic disks, received diagnosis by general ophthalmologists and were not referred. In addition, most patients included in the study were white and, as blacks are at a greater risk of developing POAG, this could have also led to the low prevalence of POAG in our sample.

We look with great concern that despite NTG being the most prevalent glaucoma in our population, the patients had more advanced disease on average. They were older, had greater *C/D* ratio, thinner OCT cRNFL, and worse VF MD and VFI. This is probably related to the fact that because their IOPs were within the “normal” range, their diagnosis was delayed.

Although the Goldman applanation tonometer (GAT) is considered the gold standard for IOP measurement, in our center, we widely use Perkins tonometer, which has been proved to have accurate measurements that are closely comparable with those by GAT^(19)^.

This study has some limitations. Despite the fact that the patients were seen multiple times during the 2-year study period, owing to the difficulty in acquiring reliable examination, this paper only shows the results of the most recent and reliable information used to confirm or discard the glaucoma diagnosis. VF testing was the most difficult examination for patients and some of them did not obtain reliable results even after multiple tries, and this may have affected our ability to establish the diagnosis. Moreover, owing to the short follow-up period, we considered at least three measurements of IOP to define normal tension or hypertension; hence, some patients might have been misclassified.

Some limitations were regarding technology. At the beginning of the study, no macular ganglion cell analysis software was available at our center. Despite the lack of this information, we believe that we were still able to correctly diagnose our patients because as reported by Lisboa et al.^(17)^, spectral domain OCT RNFL measurements performed better than ONH and macular measurements for detecting preperimetric glaucomatous damage in a cohort of glaucoma suspects. In addition, the importance of central 10-2 VF testing in glaucoma suspects was acknowledged only later in 2017. As we started our study in 2016, most of our patients were not tested; thus, we could hypothesize that we may have misdiagnosed some glaucoma suspects^(8)^.

By studying this sample of glaucoma suspects, we realized that NTG patients receive a late diagnosis, often with irreversible deterioration of their VFs. It seems to us that this might be the case in multiple centers in Brazil and worldwide. In the past years, we have been seeing a great effort from well-known ophthalmologists and glaucoma societies to teach and show the importance of the optic nerve evaluation, despite intraocular pressure levels, to detect GON earlier. Susanna et al. have been, in the past 15 years, widely promoting education about the “five rules to evaluate the optic disk and RNFL for glaucoma”^(18)^ to guide ophthalmologists to correctly interpret the findings of the optic nerve examination, thus increasing early detection of glaucoma despite IOP levels. As glaucoma usually progresses slowly, we hope that younger ophthalmologists, who have been influenced by the knowledge that observing the optic nerve is critical for glaucoma diagnosis, will diagnose NTG and other optic neuropathies earlier.
